# Root canal morphology of permanent teeth in a Malaysian subpopulation using cone-beam computed tomography

**DOI:** 10.1186/s12903-019-0710-z

**Published:** 2019-01-14

**Authors:** Julia Yen Yee Pan, Abhishek Parolia, Siong Ren Chuah, Shekhar Bhatia, Sunil Mutalik, Allan Pau

**Affiliations:** 1 School of Dentistry, International Medical University, Bukit Jalil, Kuala Lumpur, Malaysia; 2 Division of Clinical Dentistry, School of Dentistry, International Medical University (IMU), Bukit Jalil, Kuala Lumpur, Malaysia; 3 Section of Oral and Maxillofacial Radiology, School of Dental Medicine, University of Connecticut Health Center, Farmington, CT USA; 4 Dean, School of Dentistry, International Medical University, Bukit Jalil, Kuala Lumpur, Malaysia; 5 Bukit Jalil, Kuala Lumpur, Malaysia

**Keywords:** Cone-beam computed tomography, Malaysian, Morphology, Root canal

## Abstract

**Background:**

To determine the root canal morphology of human permanent maxillary and mandibular teeth in a Malaysian subpopulation using cone-beam computed tomography (CBCT).

**Methods:**

A total of 208 CBCT images were examined retrospectively. Prevalence of an extra root/canal and internal morphology based on Vertucci’s classification were observed in human maxillary and mandibular permanent teeth. Variations in the external and internal morphology were compared in relation to gender and tooth side (left vs right) using Pearson Chi-square and Fisher’s exact tests with significance level set at *p* < 0.05.

**Results:**

In the maxillary arch, the prevalence of three canals were observed in 0.3% of first premolars and two canals in 46.5% of second premolars. Males displayed significantly higher prevalence of two canals in maxillary second premolars than females (*p* < 0.05). The prevalence of a second mesiobuccal canal in maxillary first and second molars were 36.3 and 8.5%, respectively. Males displayed significantly higher prevalence of a second mesiobuccal canal in maxillary second molars than females (*p* < 0.05). The prevalence of a second palatal canal in maxillary first and second molars were 0.9 and 0.6%, respectively. In the mandibular arch, the prevalence of two canals were observed in 5.1% of central incisors, 12.3% of lateral incisors, 6.1% of canines, 18.7% of first premolars and 0.5% of second premolars. The prevalence of a middle mesial canal, second distal canal and extra root (radix entomolaris) were detected in 1.9, 19.5 and 21.4% of mandibular first molars, respectively. The prevalence of a C-shaped canal was observed in 48.7% of mandibular second molars. Females displayed significantly higher prevalence of a C-shaped canal in the right mandibular second molars than males (*p* < 0.05). No other statistically significant differences in root anatomy and root canal morphology were observed in relation to gender and tooth side.

**Conclusions:**

Wide variations in the root canal morphology exist among Malaysians. CBCT is a clinically useful tool in the identification of external and internal morphological variations in the human teeth.

## Background

The success of an endodontic treatment requires a comprehensive knowledge of the root anatomy and root canal morphology along with its variations within the norm. In dental practice, the presence of anatomical aberrations in the root and root canal system may pose a major endodontic challenge to clinicians. Therefore, thorough knowledge and understanding of their anatomy and variations is of utmost importance to avoid potential complications and undesirable failures during endodontic procedures [1].

Globally, there are numerous studies [2–7] reporting the internal and external morphological variations among different ethnicities. Only a few studies [8–11] had been conducted to evaluate the root canal morphology in a Malaysian population.

Abd Latib et al. [8] reported the prevalence of a second mesiobuccal canal in maxillary first molars among Malaysians was 18% for males and 10% for females. Choi et al. [9] compared the incidence of a second mesiobuccal canal in maxillary first molars between Caucasians and Malaysians. In that study, 65.44% of Malaysians were found to have two mesiobuccal canals [9]. Bhatia et al. [10] reported the prevalence of radix molars in extracted first molars among Malaysians was 4.9% whereby the prevalence of radix entomolaris (4.2%) was more common than radix paramolaris (0.7%). Besides that, an in vitro study by Nie et al. [11] reported that the occurrence of C-shaped canal in mandibular second molars among Malaysians was 8 (3.3%) out of 241 cases or 88.8% of total number of mandibular molars. In that study, C-shaped canal was found to be more common in the Chinese race in which it was proven to be statistically significant [11].

Various methodologies have been employed to evaluate the root and root canal morphology such as the use of clearing technique, cross-sectioning technique, analysis of extracted teeth, conventional radiography and cone-beam computed tomography (CBCT). With the advent of CBCT, many recent endodontic studies have been incorporating the three-dimensional approach as CBCT offers a wide array of advantages. CBCT permits modification of the visual field, yields a high resolution and produces a minimal amount of radiation as compared to conventional computed tomography [12]. Furthermore, majority of the CBCT equipment are ergonomically designed for safe use and optimal performance [12].

The present study therefore aimed to investigate the prevalence of morphological variations in the root anatomy and root canal morphology of human maxillary and mandibular permanent teeth in a Malaysian subpopulation using CBCT and to correlate the findings with gender and tooth side.

## Methods

### Sample selection

The present study consisted of 208 CBCT images of maxillary and mandibular permanent teeth that had been taken from patients who visited the Oral Health Centre at International Medical University (IMU) for diagnosis and preoperative assessment for implants, surgical extraction and orthodontic treatment. Written informed consents were obtained from patients whose images were used. Based on the previous studies [8–11] on Malaysian population, an expected frequency of 15%, an acceptable margin of error of 5% and a design effect set at 1 with 95% confidence interval were used as parameters for Epi Info StatCalc statistical software (version 7.2.0.1) to calculate the total sample size. Epi Info is a trademark of the Centers for Disease Control and Prevention.

### Inclusion and exclusion criteria

Healthy, untreated, fully developed, permanent maxillary and mandibular incisors, canines, first and second premolars and molars in individuals were included. Cases whereby anatomy was compromised by physiological or pathological processes and the original root anatomy was not clear, were excluded.

### Pilot study

Prior to the start of study, the researcher underwent training from trained oral radiologist and endodontists on how to operate the CBCT and identify root canal morphology from CBCT images, respectively. The pilot study was carried out using 20 CBCT images whereby all observations were performed independently by the researcher, oral radiologist and endodontists. CBCT images were studied using the same computer and screen under ambient room lighting conditions. Inter-observer reliability test was carried out using Cohen’s Kappa test (< 0 less than chance agreement, 0.01–0.20 slight agreement, 0.21–0.40 fair agreement, 0.41–0.60 moderate agreement, 0.61–0.80 substantial agreement, and 0.81–0.99 almost perfect agreement) as proposed by Viera & Garrett [13]. A final consensus was reached when the inter-rater agreement kappa value was found to be within the substantial agreement (0.61–0.80) or almost perfect agreement (0.81–099). Throughout the study, the calibration of the researcher was tested by repeating the observations twice on a random sample of 50 CBCT images.

### Radiographic technique and image analysis

The images for the study were selected from the database of KaVo 3D eXam imaging system (Imaging Sciences International, Hatfield, PA, USA). The images were acquired from a standard protocol for patient positioning, exposure parameter setting (120 kVp, 5 mA, 26.9 s) and image acquisition of 0.25 mm voxel size. The images were analysed on eXam Vision software version 1.9.3.13 (KaVo Dental GmbH, Biberach, Germany). The axial, coronal and sagittal planes, as well as custom planes of the selected images were magnified to 150% to visualise the cross section of root canal morphology.

### Measurements

The variables evaluated were patient’s gender, age, ethnicity, tooth side, number of roots and root canal configuration. The canal configurations were categorized into the following eight types based on Vertucci’s classification [14]:

1. Type I: A single canal extends from the pulp chamber to the apex.
2. Type II: Two separate canals leave the pulp chamber and join short of the apex to form one canal.
3. Type III: One canal leaves the pulp chamber and divides into two within the root, and then merge to exit as one canal.
4. Type IV: Two separate and distinct canals extend from the pulp chamber to the apex.
5. Type V: One canal leaves the pulp chamber and divides short of the apex into two separate and distinct canals with separate apical foramina.
6. Type VI: Two separate canals leave the pulp chamber, merge in the body of the root, and redivide short of the apex to exit as two distinct canals.
7. Type VII: One canal leaves the pulp chamber, divides and then reunite within the body of the root, and finally redivides into two distinct canals short of the apex.
8. Type VIII: Three separate and distinct canals extend from the pulp chamber to the apex.

The maxillary teeth were observed radiographically for (i) frequency of two canals in maxillary central incisors, lateral incisors and canines, (ii) frequency of three canals in maxillary first premolars, (iii) frequency of two canals in maxillary second premolars and (iv) frequency of second mesiobuccal and second palatal canal in maxillary first and second molars. On the other hand, the mandibular teeth were observed radiographically for (v) frequency of two canals in mandibular central incisors, lateral incisors, canines, first premolars and second premolars, (vi) frequency of middle mesial canal, second distal canal and extra root in mandibular first molars and (vii) frequency of C-shaped canal in mandibular second molars.

### Statistical analysis

Data were tabulated and analysed using IBM SPSS Statistics version 18.0 (IBM Co., Chicago, IL, USA). Pearson Chi-square test and Fisher’s exact test were employed to compare the findings with respect to gender and tooth side. The significance level was set at *p* < 0.05 in all cases.

## Results

A total of 208 Malaysians participated in the present study of which 90 (43.3%) were males and 118 (56.7%) were females within the age range of 15–66 years (mean age of 28.7 years). Majority were Chinese (92.3%) followed by Indians (4.3%), Malays (2.4%) and other races (1.0%). A total of 2448 maxillary teeth (347 central incisors, 362 lateral incisors, 404 canines, 304 first premolars, 333 s premolars, 344 first molars, 354 s molars) and 2723 mandibular teeth (408 central incisors, 400 lateral incisors, 411 canines, 359 first premolars, 399 s premolars, 370 first molars and 376 s molars) were analysed.

### Maxillary teeth

**Fig. 1:**
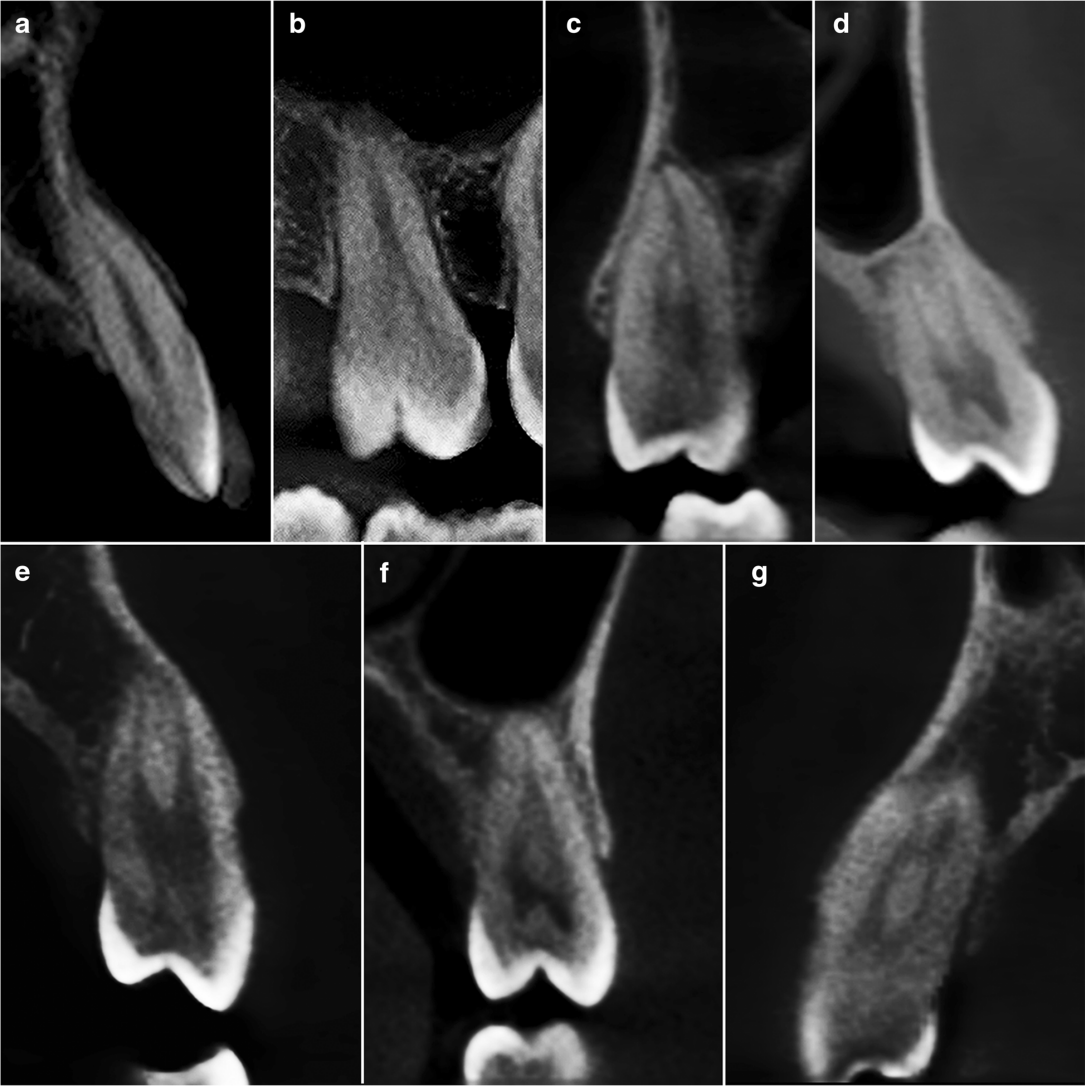
CBCT images of maxillary teeth in custom planes (reconstructed based on axial, sagittal and coronal planes) based on Vertucci’s classification. **a** Type I – right maxillary central incisor (**b**) Type II – right maxillary first molar (**c**) Type III – right maxillary second premolar (**d**) Type IV – left maxillary first premolar (**e**) Type V – left maxillary first premolar (**f**) Type VI – left maxillary second premolar (**g**) Type VII – right maxillary first premolar

The distribution of the root canal morphology of maxillary teeth based on Vertucci’s classification are shown in Table 1. Based on Fig. 1(a-g), Type I, II, III, IV, V, VI and VII configurations were reported among maxillary teeth.

**Table 1:** Root anatomy and root canal morphology of maxillary teeth based on Vertucci’s classification

Maxillary teeth	Number of roots	Root Type	Vertucci’s classification							
			Type I	Type II	Type III	Type IV	Type V	Type VI	Type VII	Type VIII
Central Incisor (*n* = 347)	1	–	347 (100.0%)	–	–	–	–	–	–	–
Lateral Incisor (*n* = 362)	1	–	362 (100.0%)	–	–	–	–	–	–	–
Canine (*n* = 404)	1	–	404 (100.0%)	–	–	–	–	–	–	–
First Premolar (*n* = 304)	1 (*n* = 206)	–	35 (17.0%)	49 (23.8%)	32 (15.5%)	46 (22.3%)	11 (5.3%)	28 (13.6%)	5 (2.4%)	–
	2 (*n* = 97)	Buccal	97 (100.0%)	–	–	–	–	–	–	–
		Palatal	97 (100.0%)	–	–	–	–	–	–	–
	3 (*n* = 1)	Mesial	1 (100.0%)	–	–	–	–	–	–	–
		Distal	1 (100.0%)	–	–	–	–	–	–	–
		Palatal	1 (100.0%)	–	–	–	–	–	–	–
Second Premolar (*n* = 333)	1 (*n* = 306)	–	178 (58.2%)	60 (19.6%)	31 (10.1%)	6 (2.0%)	20 (6.5%)	10 (3.3%)	1 (0.3%)	–
	2 (*n* = 27)	Buccal	26 (96.3%)	–	–	–	1 (3.7%)	–	–	–
		Palatal	26 (96.3%)	–	–	–	–	–	–	–
First Molar (*n* = 344)	2 (*n* = 1)	Buccal	1 (100.0%)	–	–	–	–	–	–	–
		Palatal	1 (100.0%)	–	–	–	–	–	–	–
	3 (*n* = 343)	Mesiobuccal	219 (63.8%)	15 (4.4%)	5 (1.5%)	99 (28.9%)	5 (1.5%)	–	–	–
		Distobuccal	343 (100.0%)	–	–	–	–	–	–	–
		Palatal	340 (99.1%)	3 (0.9%)	–	–	–	–	–	–
Second Molar (*n* = 354)	1 (*n* = 10)	–	4 (40.0%)	2 (20.0%)	–	–	–	–	–	4 (40.0%)
	2 (*n* = 32)	Buccal	25 (78.1%)	4 (12.5%)	–	3 (9.4%)	–	–	–	–
		Palatal	30 (93.8%)	2 (6.2%)	–	–	–	–	–	–
	3 (*n* = 312)	Mesiobuccal	282 (90.4%)	6 (1.9%)	–	24 (7.7%)	–	–	–	–
		Distobuccal	312 (100.0%)	–	–	–	–	–	–	–
		Palatal	312 (100.0%)	–	–	–	–	–	–	–

All (100%) central incisors, lateral incisors and canines exhibited type I configuration.

Majority of the first premolars were found to have one root (67.8%) followed by two roots (31.9%) which displayed bifurcation in the apical third to half of the root. Majority of the first premolars (88.2%) displayed two canals.

Majority of the second premolars (91.9%) were single-rooted with type 1 (58.2%) as the most common canal configuration. Most two-rooted second premolars demonstrated type I configuration (96.3%) in each root. Among the two-rooted second premolars, only one (3.7%) displayed a type V configuration in the buccal root.

In the first and second molars, majority of them presented with three roots in which type I configuration was the most common canal configuration for mesiobuccal root, distobuccal root and palatal root. Only one first molar exhibited two roots in which each root displayed a type I configuration. Second molars with one (2.8%) and two roots (9.0%) have also been reported.

### Maxillary central incisors, lateral incisors and canines

**Fig. 2:**
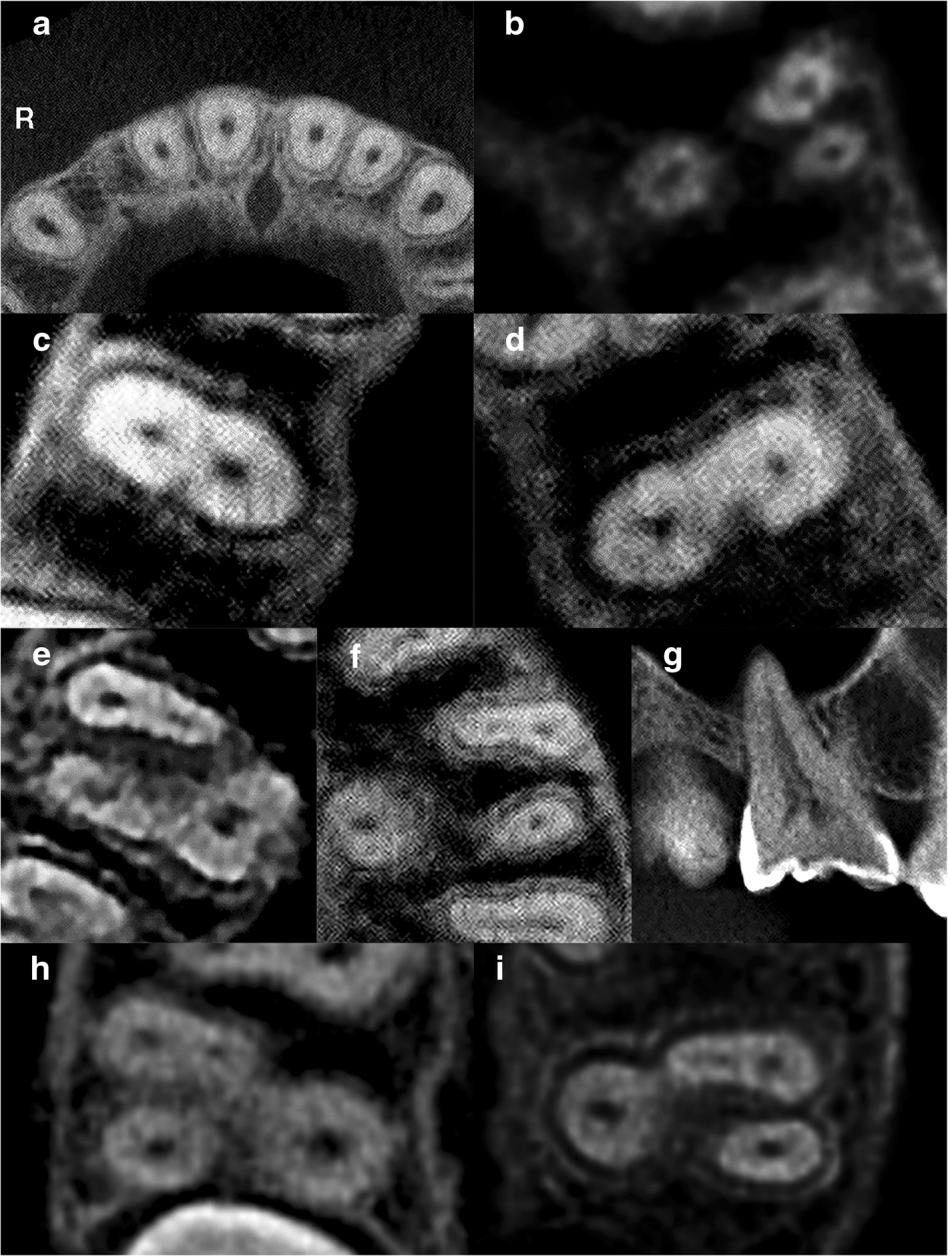
CBCT images of maxillary teeth. R – Right. **a** Axial slice of maxillary central and lateral incisors and canines displaying single canals (**b**) Axial slice of a three-rooted left maxillary first premolar displaying one canal in each root. **c** Axial slice of a right maxillary second premolar displaying two canals (**d**) Axial slice of a left maxillary second premolar displaying two canals. **e** Axial slice of a right maxillary first molar displaying a second mesiobuccal (MB2) canal in its mesial root. **f** Axial slice of a left maxillary first molar displaying a second mesiobuccal (MB2) canal in its mesial root. **g** Sagittal slice of a right maxillary first molar displaying a second palatal canal. **h** Axial slice of a right maxillary second molar displaying a second mesiobuccal (MB2) canal in its mesial root. **i** Axial slice of a left maxillary second molar displaying a second mesiobuccal (MB2) canal in its mesial root

All (100%) central incisors, lateral incisors and canines exhibited a single canal. The canal configurations are shown in Fig. 2(a). No statistically significant difference in the prevalence of a second canal was observed between gender and tooth side for incisors and canines (*p* > 0.05).

### Maxillary first premolars

The prevalence of three canals in first premolars was 0.3% (Table 2). As shown in Fig. 2(b), this unusual occurrence was detected in a three-rooted first premolar in which each root encases a single root canal from the orifice to the apex.

**Table 2:** Root anatomy and root canal morphology of maxillary first and second premolars according to gender and tooth side

	Maxillary first premolar (*n* = 304)				Maxillary second premolar (*n* = 333)		
	One Root		Two Roots	Three Roots	One Root		Two Roots
	One Canal	Two Canals	Two Canals	Three Canals	One Canal	Two Canals	Two Canals
Left	16 (10.5%)	86 (56.6%)	49 (32.2%)	1 (0.7%)	87 (52.4%)	66 (39.8%)	13 (7.8%)
Male	3 (2.0%)	37 (24.3%)	26 (17.1%)	1 (0.7%)	24 (14.5%)	38 (22.9%)	8 (4.8%)
Female	13 (8.6%)	49 (32.2%)	23 (15.1%)	–	63 (38.0%)	28 (16.9%)	5 (3.0%)
Right	19 (12.5%)	85 (55.9%)	48 (31.6%)	–	91 (54.5%)	62 (37.1%)	14 (8.4%)
Male	4 (2.6%)	35 (23.0%)	28 (18.4%)	–	23 (13.8%)	37 (22.2%)	10 (6.0%)
Female	15 (9.9%)	50 (32.9%)	20 (13.2%)	–	68 (40.7%)	25 (15.0%)	4 (2.4%)
Total	35 (11.5%)	171 (56.3%)	97 (31.9%)	*1* *(0.3%)*	178 (53.5%)	128 (38.4%)	27 (8.1%)
						*155* *(46.5%)*	

### Maxillary second premolars

The prevalence of two canals in second premolars was 46.5% (Table 2). The canal configurations are shown in Fig. 2(c-d). The prevalence of two canals on the left (27.7%) and right (28.1%) sides in males were found to be statistically significantly higher than females (*p* < 0.05). Overall, the prevalence of two canals in maxillary second premolars was statistically significantly higher in males (66.4%) than females (32.1%) (*p* < 0.05). No statistically significant difference was observed between the prevalence of two canals in the right and left sides (*p* > 0.05).

### Maxillary molars

The prevalence of a second mesiobuccal canal was 36.3% in first molars and 8.5% for second molars (Table 3). The canal configurations of first molars are shown in Fig. 2(e-g). In the right second molars, the prevalence of a second mesiobuccal canal was found to be statistically significantly higher in males (6.2%) (*p* < 0.05). Overall, the prevalence of a second mesiobuccal canal in second molars was statistically significantly higher in males (13.4%) than females (4.9%) (*p* < 0.05). No statistically significant difference was observed between the prevalence of a second mesiobuccal canal in the right and left sides (*p* > 0.05).

**Table 3:** Root anatomy and root canal morphology of maxillary first and second molars according to gender and tooth side

	Maxillary First Molar (*n* = 344)				Maxillary Second Molar (*n* = 354)			
	Second Mesiobuccal Canal		Second Palatal Canal		Second Mesiobuccal Canal		Second Palatal Canal	
	Present	Absent	Present	Absent	Present	Absent	Present	Absent
Left	62 (36.0%)	110 (64.0%)	1 (0.6%)	171 (99.4%)	16 (9.0%)	161 (91.0%)	1 (0.6%)	176 (99.4%)
Male	28 (16.3%)	42 (24.4%)	–	70 (40.7%)	9 (5.1%)	64 (36.2%)	–	73 (41.2%)
Female	34 (19.8%)	68 (39.5%)	1 (0.6%)	101 (58.7%)	7 (4.0%)	97 (54.8%)	1 (0.6%)	103 (58.2%)
Right	63 (36.6%)	109 (63.4%)	2 (1.2%)	170 (98.8%)	14 (7.9%)	163 (92.1%)	1 (0.6%)	176 (99.4%)
Male	30 (17.4%)	42 (24.4%)	1 (0.6%)	71 (41.3%)	11 (6.2%)	65 (36.7%)	–	76 (42.9%)
Female	33 (19.2%)	67 (39.0%)	1 (0.6%)	99 (57.6%)	3 (1.7%)	98 (55.4%)	1 (0.6%)	100 (56.5%)
Total	*125 (36.3%)*	219 (63.7%)	*3 (0.9%)*	341 (99.1%)	*30 (8.5%)*	324 (91.5%)	*2 (0.6%)*	352 (99.4%)

The prevalence of a second palatal canal was also found in 0.9% of first molars and 0.6% of second molars (Table 3). The canal configurations of second molars are shown in Fig. 2 (h-i). No statistically significant difference in the prevalence of a second palatal canal was observed between gender and tooth side (*p* > 0.05).

### Mandibular teeth

**Fig. 3:**
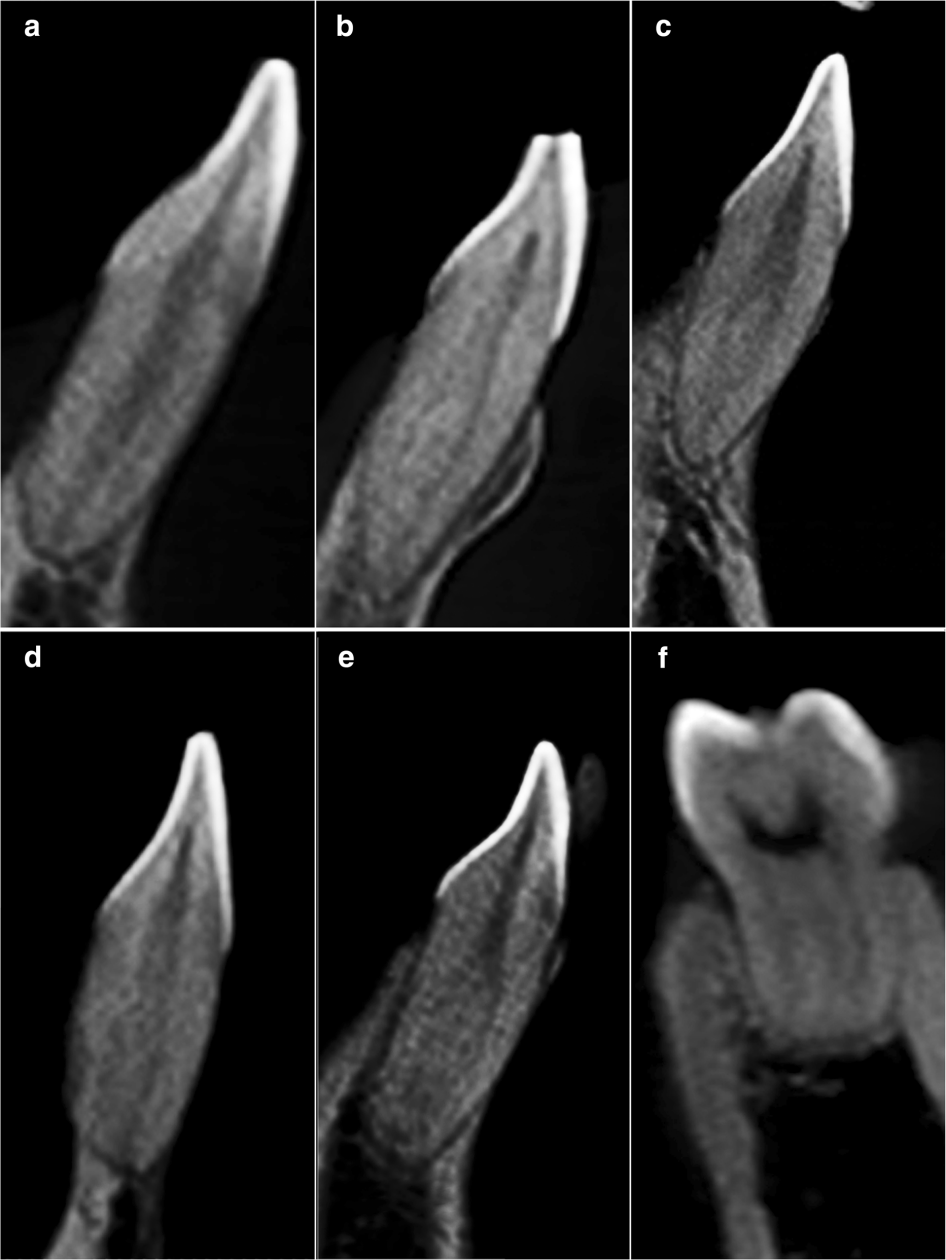
CBCT images of mandibular teeth in custom planes (reconstructed based on axial, sagittal and coronal planes) based on Vertucci’s classification. **a** Type I – left mandibular central incisor (**b**) Type II – right mandibular central incisor (**c**) Type III – left mandibular lateral incisor (**d**) Type IV – left mandibular central incisor (**e**) Type V – right mandibular left lateral incisor (**f**) Type VIII – left mandibular first molar

The distribution of the root canal morphology of mandibular teeth based on Vertucci’s classification are shown in Table 4. Based on Fig. 3(a-f), Type I, II, III, IV, V and VIII configurations were reported among mandibular teeth.

**Table 4:** Root anatomy and root canal morphology of mandibular teeth based on Vertucci’s classification

Mandibular Teeth	Number of Roots	Root Type	Vertucci’s Classification							
			Type I	Type II	Type III	Type IV	Type V	Type VI	Type VII	Type VIII
Central Incisor (*n* = 408)	1	–	387 (94.9%)	–	4 (1.0%)	–	17 (4.2%)	–	–	–
Lateral Incisor (*n* = 400)	1	–	351 (87.8%)	–	15 (3.8%)	1 (0.3%)	33 (8.3%)	–	–	–
Canine (*n* = 411)	1 (*n* = 406)	–	386 (95.1%)	20 (4.9%)	–	–	–	–	–	–
	2 (*n* = 5)	Buccal	5 (100.0%)	–	–	–	–	–	–	–
		Palatal	5 (100.0%)	–	–	–	–	–	–	–
First Premolar (*n* = 359)	1 (*n* = 353)	–	292 (82.7%)	1 (0.3%)	5 (1.4%)	–	55 (15.6%)	–	–	–
	2 (*n* = 6)	Mesial	6 (100.0%)	–	–	–	–	–	–	–
		Distal	3 (50.0%)	–	–	3 (50.0%)	–	–	–	–
Second Premolar (*n* = 399)	1	–	397 (99.5%)	1 (0.3%)	–	1 (0.3%)	–	–	–	–
First Molar (*n* = 370)	2 (*n* = 290)	Mesial	7 (2.4%)	6 (2.1%)	–	270 (93.1%)	–	–	–	7 (2.4%)
		Distal	220 (75.9%)	6 (2.1%)	–	63 (21.7%)	1 (0.3%)	–	–	–
	3 (*n* = 80)	Mesial	2 (2.5%)	–	–	78 (97.5%)	–	–	–	–
		Distobuccal	42 (52.5%)	–	1 (1.3%)	–	–	–	–	–
		Distolingual	79 (98.8%)	–	–	–	1 (1.3%)	–	–	–
Second Molar (*n* = 376)	2 (*n* = 192)	Mesial	34 (17.7%)	8 (4.2%)	–	150 (78.1%)	–	–	–	–
		Distal	186 (96.9%)	–	–	6 (3.1%)	–	–	–	–
	3 (*n* = 1)	Mesial	–	–	–	1 (100.0%)	–	–	–	–
		Distobuccal	1 (100.0%)	–	–	–	–	–	–	–
		Distolingual	1 (100.0%)	–	–	–	–	–	–	–
		C-shape	183 (48.7%)	–	–	–	–	–	–	–

Majority of the central (94.9%) and lateral incisors (87.8%) exhibited type I configuration.

Majority of the canines were single-rooted (98.8%) with type I (95.1%) and type II (4.9%) configurations.

In the first premolars, majority of them were single-rooted (98.3%) with type I (82.7%) as the most common canal configuration.

In the second premolars, all (100%) were displayed as single-rooted with type I (99.5%) as the most prevalent canal configuration.

Most of the first molars were two-rooted (78.4%) in which type IV (93.1%) and type I (75.9%) were the most common canal configuration in the mesial and distal roots, respectively. In the first molars with additional distolingual root (radix entomolaris), type I (98.8%) configuration was most prevalent. Majority of the second molars were two-rooted (51.1%) followed by C-shaped configuration (48.7%). In the two-rooted second molars, type IV (78.1%) and type I (96.9%) were the most common canal configuration in the mesial and distal roots, respectively.

### Mandibular central incisors, lateral incisors and canines

**Fig. 4:**
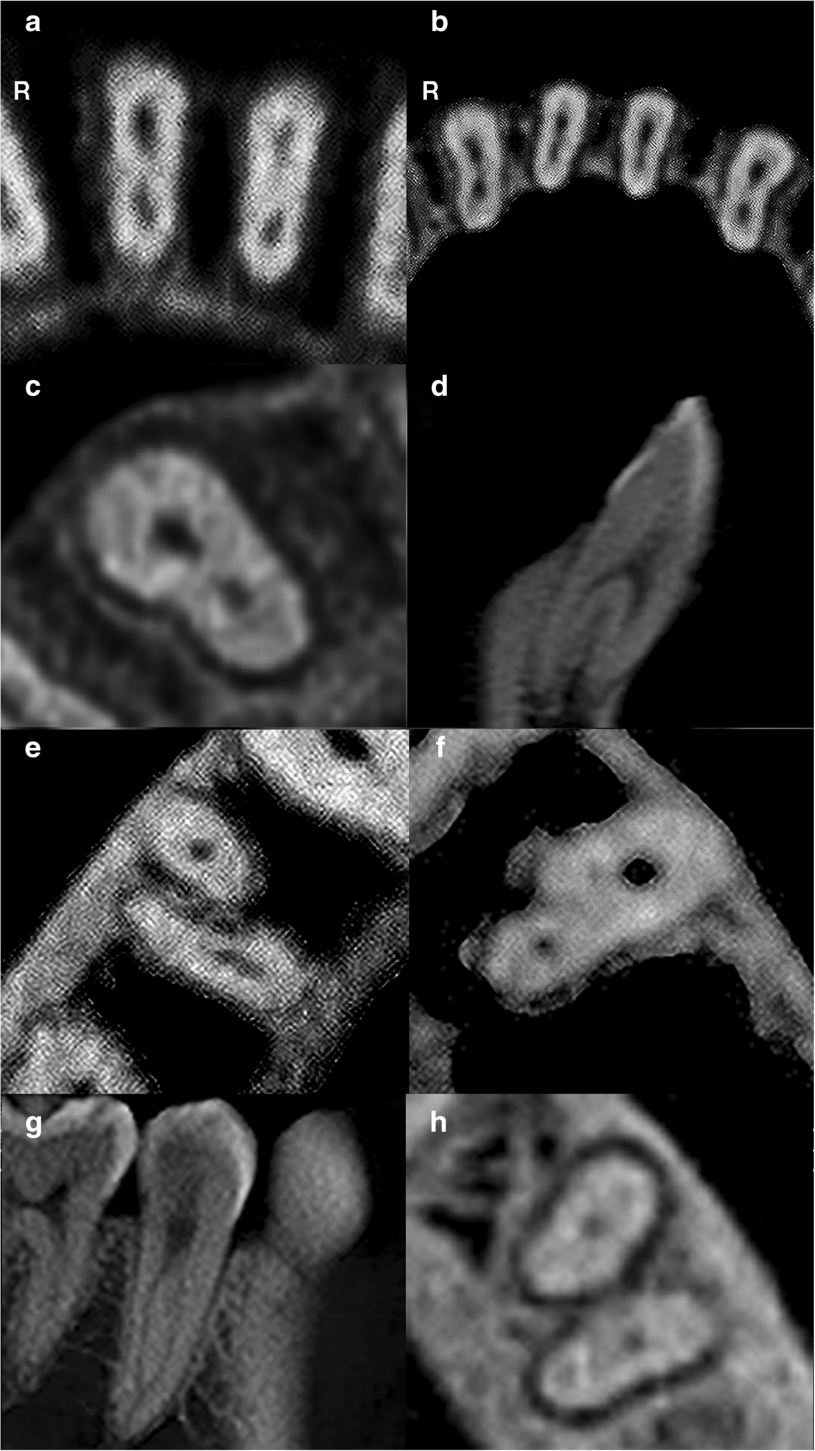
CBCT images of mandibular teeth. R – Right. **a** Axial slice of mandibular central incisors displaying two canals. **b** Axial slice of mandibular lateral incisors displaying two canals and canines displaying one canal. **c** Axial slice of a right mandibular canine displaying two canals. **d** Sagittal slice of a two-rooted left mandibular canine displaying two canals. **e** Axial slice of a two-rooted right mandibular first premolar displaying one canal in each root. **f** Axial slice of a left mandibular first premolar displaying two canals. **g** Sagittal slice of a single-rooted right mandibular second premolar displaying two canals. **h** Axial slice of a two-rooted left mandibular second premolar displaying one canal in each root

The prevalence of two canals in central incisors, lateral incisors and canines were 5.1, 12.3 and 6.1%, respectively. Two-rooted canines (1.2%) were also reported. The canal configurations are shown in Fig. 4(a-d). No statistically significant difference in the prevalence of two canals was observed between gender and tooth side for incisors and canines (*p* > 0.05).

### Mandibular first premolars

The prevalence of two canals in first premolars was 18.7%. The canal configurations are shown in Fig. 4(e-f). No statistically significant difference in the prevalence of two canals was observed between gender and tooth side for first premolars (*p* > 0.05).

### Mandibular second premolars

The prevalence of two canals in second premolars was 0.5%. The canal configurations are shown in Fig. 4(g-h). No statistically significant difference in the prevalence of two canals was observed between gender and tooth side for second premolars (*p* > 0.05).

### Mandibular first molars

**Fig. 5:**
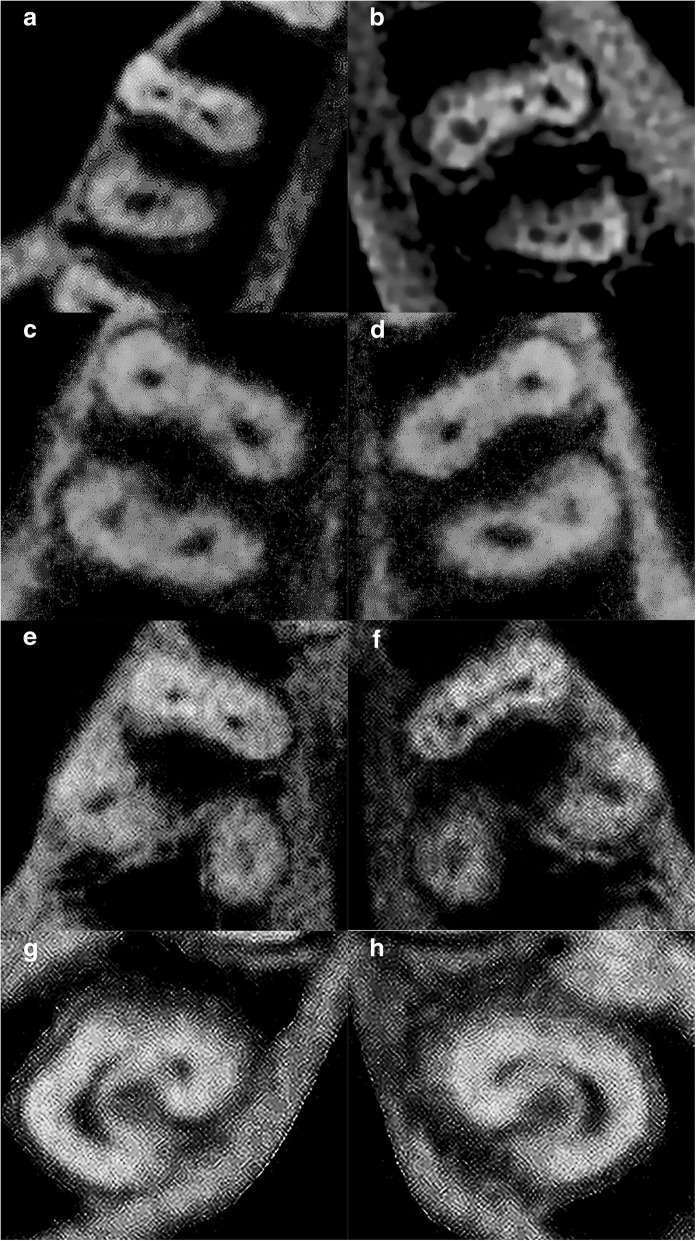
CBCT images of mandibular teeth. **a** Axial slice of a right mandibular first molar displaying a middle mesial canal in its mesial root. **b** Axial slice of a left mandibular first molar displaying a middle mesial canal in its mesial root. **c** Axial slice of a right mandibular first molar displaying a second distal canal in its distal root. **d** Axial slice of a left mandibular first molar displaying a second distal canal in its distal root. **e** Axial slice of a right mandibular first molar displaying an extra distolingual root (radix entomolaris). **f** Axial slice of a left mandibular first molar displaying an extra distolingual root (radix entomolaris). **g** Axial slice of a right mandibular second molar displaying a C-shaped canal. **h** Axial slice of a left mandibular second molar displaying a C-shaped canal

The prevalence of a middle mesial canal and second distal canal in first molars were 1.9 and 19.5%, respectively. (Table 5). On the other hand, the prevalence of radix first molars was 21.6% in which all were identified as radix entomolaris (extra distolingual root) (Table 5). All canal and root configurations are shown in Fig. 5(a-f). No statistically significant difference in the prevalence of a middle mesial canal, second distal canal and radix molar were observed between gender and tooth side for first molars (*p* > 0.05).

**Table 5:** Root canal morphology of mandibular first molars according to gender and tooth side

	Mandibular First Molar (*n* = 370)							
	Middle mesial canal		Second distal canal		Two roots	Three roots		
	Present	Absent	Present	Absent		Radix entomolaris	Radix paramolaris	Mesial root bifurcation
Left	2 (1.1%)	184 (98.9%)	39 (21.0%)	147 (79.0%)	149 (80.1%)	36 (19.4%)	–	1 (0.5%)
Male	0 (0.0%)	77 (41.4%)	19 (10.2%)	58 (31.2%)	62 (33.3%)	14 (7.5%)	–	1 (0.5%)
Female	2 (1.1%)	107 (57.5%)	20 (10.8%)	89 (47.8%)	87 (46.8%)	22 (11.8%)	–	–
Right	5 (2.7%)	179 (97.3%)	33 (17.9%)	151 (82.1%)	141 (76.6%)	43 (23.4%)	–	–
Male	1 (0.5%)	76 (41.3%)	15 (8.2%)	62 (33.7%)	59 (32.1%)	18 (9.8%)	–	–
Female	4 (2.2%)	103 (56.0%)	18 (9.8%)	89 (48.4%)	82 (44.6%)	25 (13.6%)	–	–
Total	*7 (1.9%)*	363 (98.1%)	*72 (19.5%)*	298 (80.5%)	290 (78.4%)	*79 (21.4%)*	–	1 (0.3%)

### Mandibular second molars

The prevalence of a C-shaped canal in second molars was 48.7% (Table 6). The C-shaped configuration are shown in Fig. 5(g-h). In the right second molars, the prevalence of a C-shaped canal was found to be statistically significantly higher in females (31.4%) (*p* < 0.05). Overall, the prevalence of a C-shaped canal in second molars was higher in females (54.2%) than males (41.3%) (*p* < 0.05).

**Table 6:** Root canal morphology of mandibular second molars according to gender and tooth side

	Mandibular second molar (*n* = 376)	
	C-Shaped Canal	
	Present	Absent
Left	94 (49.2%)	97 (50.8%)
Male	35 (18.3%)	46 (24.1%)
Female	59 (30.9%)	51 (26.7%)
Right	89 (48.1%)	96 (51.9%)
Male	31 (16.8%)	48 (25.9%)
Female	58 (31.4%)	48 (25.9%)
Total	*183 (48.7%)*	193 (51.3%)

## Discussion

The present study has shown vast anatomical variations in the root and root canal morphology of human maxillary and mandibular permanent teeth in a Malaysian subpopulation. Considering that the anatomical features of root canals influence the outcome of a root canal treatment, clinicians must be attentive to the anatomic complexities present. Iatrogenic procedural errors such as missed canals, perforations or canal transportations arise from an inadequate knowledge of root canal morphology.

The application of CBCT in clinical dental practice and studies has risen in popularity in recent years. With its non-invasive approach, CBCT aids clinicians in diagnosing an endodontic problem and formulating an accurate treatment plan. CBCT has been shown to be reliable and promising in detecting root canal anatomy when compared to gold standard of visual inspection through physically sectioning the tooth [15]. According to the American Association of Endodontists and American Academy of Oral and Maxillofacial Radiology Joint Position Statement published in 2015 [16], clinicians are advised to employ CBCT only when lower dose conventional dental radiography or other imaging modalities fails to adequately capture the images. Generally, clinicians predetermine and target a small area of interest in endodontic cases [16]. In endodontic cases deemed appropriate for the use of CBCT, a smaller field of view which is associated with lower radiation dose and high-resolution images, is recommended when establishing the primary diagnosis [16].

The present study detected a single canal in 100% of maxillary central incisors, lateral incisors and canines which is consistent with clearing and radiographic studies by Vertucci [14], Pineda and Kuttler [17] and Kasahara et al. [18] in the Caucasian, Mexican and Japanese populations, respectively. Martins et al. [19] also detected a single canal in all maxillary incisors and canines in both Asians and Caucasians. On the other hand, the incidence of two canals in maxillary incisors and canines were reported in clearing studies by Sert and Bayirli [2] and Weng et al. [20] in the Turkish and Chinese populations, respectively. Variations in the number of canals in maxillary incisors and canines may be due to differences in geographic areas. The incidence of two or more canals in maxillary incisors and canines are known to be extremely rare. In such uncommon cases, anomalies such as fusion, germination, dens invaginatus and talon cusps must be considered in the maxillary incisors and canines [1].

Maxillary first premolar has a very complex root canal anatomy due to its variation in the number of roots and canals which can range from a single root to three roots and from a single canal to three canals, respectively [1]. The presence of root concavities on both mesial and distal surfaces of the root contributes to a kidney shape root that is broad buccolingually and narrow mesiodistally [1]. With these anatomical features, maxillary first premolars are deemed to be more susceptible to endodontic root perforations [1]. The present study detected only one (0.3%) three-rooted maxillary first premolar with three canals. Kartal et al. [21] and Gupta et al. [22] also reported three canals in 1.66 and 0.4% of maxillary first premolars in the Turkish and North Indian populations using clearing technique, respectively. Martins et al. [19] studied the maxillary first premolars of Caucasians and Asians in which the incidence of a three-rooted (4.3%) and Type VIII (0.7%) configurations were reported in the Caucasian group. In the present study, the most common canal configuration recorded for single-rooted maxillary first premolars was Type II (23.8%) followed by Type IV (22.3%). According to Martins et al. [19], Asians displayed a higher prevalence of single-rooted and Type IV (55.0%) configurations in maxillary first premolars. Vertucci [14] also recorded similar findings with Type II and IV being the most prevalent canal configurations among maxillary first premolars.

It is widely acknowledged that majority of maxillary second premolars have one root with one canal [1]. Nonetheless, a high incidence of two canals in second premolars had been reported in numerous studies [2, 14, 19, 21]. The present study demonstrated that 46.5% of maxillary second premolars had two canals whereby Martins et al. [19] and Kartal et al. [21] also recorded similar incidence in the Asian subpopulation and Turkish population, respectively. The results of the present study do not concur with Yang et al. [23] and Al-Ghananeem et al. [24] who reported a higher incidence of two canals and lower incidence of one canal in the Chinese subpopulation and Jordanian population, respectively. Differences in Asian ethnic groups may account for the variable findings which require additional research. The present study also showed that the prevalence of two canals in maxillary second premolars was statistically significantly higher in left and right of male subjects (*p* < 0.05). Overall, the prevalence of two canals in maxillary second premolars was higher in males than females (*p* < 0.05). Al-Nazhan et al. [25] also reported a significant difference in the prevalence of two root canals among maxillary second premolars between males (69.4%) and females (52.2%) in a Saudi Arabian subpopulation. Type I configuration was most commonly reported in the present study, as well as studies by Sert and Bayirli [2], Vertucci [14], Martins et al. [19] and Yang et al. [23].

Maxillary first molars are considered as one of the most frequently endodontically treated teeth due to their variations in the number of canals, which in turn may jeopardise the success of root canal treatment [1]. One of the most frequently missed canals in maxillary molars is the second mesiobuccal canal with the canal exiting the chamber at an acute mesial inclination and subsequently bending distally which makes its negotiation a challenge for clinicians [26]. The present study showed that the prevalence of a second mesiobuccal canal in maxillary first molars was 36.3% which is in accordance with CBCT studies by Abd Latib et al. [8] and Aktan et al. [27] who reported a prevalence of below 50% in the Malaysian and Iranian populations, respectively. However, this finding is at a lower prevalence rate when compared to studies by Cleghorn et al. [1], Choi et al. [9] and Martins et al. [28] which may be attributed to different sample sizes, methodologies and ethnic groups. The occurrence of a second mesiobuccal canal is also frequently encountered in maxillary second molars. The present study reported 8.5% of maxillary second molars had a second mesiobuccal canal which is comparable to the CBCT results by Tanvi et al. [29], Ghoncheh et al. [30] and Olczak et al. [31] in the Indian subpopulation, Iranian and Polish populations, respectively. However, studies stated by Cleghorn et al. [1] and Betancourt et al. [32] reported a higher prevalence of 47.1 and 48%, respectively. Martins et al. [19] found that second mesiobuccal canals in maxillary first and second molars were more frequently reported in the Caucasians than in the Asians. In the present study, the prevalence of a second mesiobuccal canal in maxillary second molars was statistically significantly higher in males (*p* < 0.05) which is similarly reported by Olczak et al. [31]. Apart from that, the present study detected the prevalence of a second palatal canal was 0.9 and 0.6% in first and second molars, respectively which are consistent with studies by Cleghorn et al. [1] and Ratanajirasut et al. [33]. Martins et al. [19] reported two palatal canals in 1.7% of maxillary first molars and 1.4% of maxillary second molars in the Caucasian group. The prevalence of a second palatal canal in maxillary molars are extremely uncommon according to a literature review by Nosrat et al. [3] and such anatomical aberration should be taken into consideration during endodontic procedures.

Mandibular incisors and canines may have two separate canals whereby majority of the canals unite and exit through a single foramen [1]. The present study observed that the prevalence of two canals in mandibular central incisors and lateral incisors were 5.1 and 12.3%, respectively. The results of the present study correspond with Zhengyan et al. [34] and Martins et al. [19] who recorded similar low prevalence rate in the Chinese population and Asian group, respectively. On the other hand, Sert and Bayirli [2] reported a higher prevalence of two canals in 68 and 63% of mandibular central and lateral incisors, respectively in a Turkish population. In the present study, the most common canal configuration in mandibular incisors was Type I which was also reported in earlier studies by Sert and Bayirli [2], Martins et al. [19] and Zhengyan et al. [34]. The present study also observed that 6.1% of mandibular canines had 2 canals of which type I and II configurations were reported. Similarly, Martins et al. [19] observed that 2.9 and 9.8% of mandibular canines had two canals with type I-V configurations reported in the Asian and Caucasian groups, respectively. Further studies need to be conducted to determine their racial predilections as the presence of additional root canal configurations in mandibular incisors and canines may be associated with certain ethnic populations.

Mandibular first premolars are among the most challenging teeth to be treated endodontically due to their diversity in root canal morphology and difficult access to a second canal [1]. The present study demonstrated that 18.7% of mandibular first premolars had two canals and this finding is supported by CBCT studies conducted by Llena et al. [12] and Shetty et al. [35] in the Spanish and South Indian populations, respectively. On the contrary, an in vitro by Sert and Bayirli [2] presented a higher prevalence of 50% for mandibular first premolars with two canals. In the present study, Type I configuration was most commonly found followed by Type V configuration. This similar finding of Vertucci canal configuration was also reported by Llena et al. [12] and Shetty et al. [35].

Mandibular second premolars have been reported to have two or more canals in several studies [2, 12, 35]. According to Cleghorn et al. [1], the second canal is commonly narrow and branches toward the lingual aspect in the middle or apical third of the main root canal. The present study observed that 0.5% of mandibular second premolars had two canals which coincides with results by Llena et al. [12] and Shetty et al. [35]. Sert and Bayirli [2] reported a higher prevalence of two canals in 29% of mandibular second premolars. In the present study, Type I and IV configurations were reported. On the other hand, Llena et al. [12] and Shetty et al. [35] have reported a wider range of variations with Type I, II, III, V and VIII configurations. The disparities observed may be influenced by the study designs (in vivo versus in vitro) or racial differences.

Generally, mandibular molars have two mesial canals and one distal canal (1). However, the mesial root can have a third canal, known as the middle mesial canal. The present study reported that the prevalence of a middle mesial canal in mandibular first molars was 1.9% which coincides with CBCT studies by Kim et al. [36] and Miloglu et al. [37] in the Korean and Turkish populations, respectively. On the other hand, Nosrat et al. [3] reported a higher prevalence of 18.6% for middle mesial canal in mandibular first molars among non-Caucasian and Caucasian groups. These findings have pointed that ethnicity plays a factor in determining the chances of locating a middle mesial canal in mandibular molars. Majority of the mandibular first molars in the present study had type IV configuration in the mesial root which was a common finding in studies by Peiris et al. [4], Ahmed et al. [7], Kim et al. [36] and Miloglu et al. [37].

The distal root of mandibular first molars may reveal two distal canals whereby the second distal canal is in the distolingual position or in a separate distolingual root. The present study recorded a prevalence of 19.5% for second distal canal in mandibular first molars which corresponds strongly to the results by Peiris et al. [4] and Martins et al. [19]. On the other hand, Ahmed et al. [7] reported a higher incidence of 59% for two canals in distal roots of mandibular first molars in a Sudanese population. Majority of the two-rooted and three-rooted mandibular first molars recorded Type I configuration in the distal root which corresponds to studies by Sert and Bayirli [2], Peiris et al. [4], Ahmed et al. [7], Kim et al. [36] and Miloglu et al. [37].

A mandibular molar with an additional third root that is located distolingually is called radix entomolaris. Whereas, an additional root at the mesiobuccal aspect is called radix paramolaris. The present study detected 21.4% of mandibular first molars were radix molars in which all were categorized as radix entomolaris (additional root located lingually or distolingual root). Yew & Chan [38] and Huang et al. [39] reported similar prevalence of 21.5 and 25.3% in Chinese and Taiwanese populations, respectively. Martins et al. [19] also reported that 3-rooted configuration in mandibular first molars was more common in Asians (25.9%) when compared to Caucasians (2.6%). On the contrary, Ahmed et al. [7] reported a lower prevalence of 3% of radix entomolaris. Walker [40] proposed that radix entomolaris is seen as a genetic trait rather than a developmental aberration.

A C-shaped root canal is presented by a fin or a web connecting individual root canals [1]. This unique anatomical configuration is very common in mandibular second molars due to their higher incidence of root fusion [1]. The present study showed that 48.7% of mandibular second molars had a C-shaped canal. These findings are consistent with studies by Zheng et al. [5] and Kim et al. [41] whereby high prevalence of a C-shaped canal was recorded in Korean and Chinese populations, respectively. Conversely, Ahmed et al. [7] reported a low incidence of 10% for C-shaped canal in a Sudanese population. Zheng et al. [5] reported no significant difference between genders in the prevalence of a C-shaped canal. However, the present study found that C-shaped canals in the right mandibular second molars were statistically significantly more prevalent in female subjects (*p* < 0.05). Overall, the present study also revealed that the prevalence of a C-shaped canal in left and right mandibular second molars was higher in females than males (*p* < 0.05). The results of the present study are consistent with two studies on Asian populations [36, 41] whereby significant differences between females and males were observed in the distribution of C-shaped canals in left and right mandibular second molars. Kim et al. [36] and Kim et al. [41] reported that the prevalence of a C-shaped canal was statistically significantly higher in 47 and 25% of female subjects, respectively. In a CBCT study by von Zuben et al. [42], the global prevalence of C-shaped canal was 13.9% with significantly higher prevalence in the population of China (44.0%) and in females (16.5%).

## Conclusion

The results of the present study revealed that human maxillary and mandibular permanent teeth in a Malaysian subpopulation do demonstrate a considerable amount of anatomic variations and abnormalities with respect to number of roots and root canals. The CBCT can be a very useful tool in identifying morphological variations in the root canal system due to its various beneficial features over two-dimensional imaging system.

## Abbreviations

AP: Abhishek Parolia
AP: Allan Pau
CBCT: Cone-beam computed tomography
IMU: International Medical University
JYYP: Julia Yen Yee Pan
SB: Shekhar Bhatia
SM: Sunil Mutalik
SRC: Siong Ren Chuah
